# Pathogenesis and Molecular Mechanisms of Anderson–Fabry Disease and Possible New Molecular Addressed Therapeutic Strategies

**DOI:** 10.3390/ijms221810088

**Published:** 2021-09-18

**Authors:** Antonino Tuttolomondo, Irene Simonetta, Renata Riolo, Federica Todaro, Tiziana Di Chiara, Salvatore Miceli, Antonio Pinto

**Affiliations:** 1 Internal Medicine and Stroke Care Ward, Department of Promoting Health, Maternal-Infant Excellence and Internal and Specialized Medicine (ProMISE) G. D’Alessandro, University of Palermo (Italy), Piazza delle Cliniche n.2, 90127 Palermo, Italy; irene.simonetta@live.it (I.S.); renatariolo.rr@gmail.com (R.R.); federicatodaro21@gmail.com (F.T.); tiziana.dichiara@unipa.it (T.D.C.); salvatore.miceli@policlinico.pa.it (S.M.); pinto@neomedia.it (A.P.); 2 Centro di Riferimento Regionale per la Cura e Diagnosi della Malattia di Anderson–Fabry, 90127 Palermo, Italy; 3 Molecular and Clinical Medicine PhD Programme, University of Palermo, 90127 Palermo, Italy

**Keywords:** Anderson–Fabry disease, globotriaosylceramide, endothelial dysfunction, podocyturia, valvular dysfunction, miR-1307-5p, miR-21-5p, miR-152-5p, miR-26a-5p, KCa3.1 activity

## Abstract

Anderson–Fabry disease (AFD) is a rare disease with an incidenceof approximately 1:117,000 male births. Lysosomal accumulation of globotriaosylceramide (Gb3) is the element characterizing Fabry disease due to a hereditary deficiency α-galactosidase A (GLA) enzyme. The accumulation of Gb3 causes lysosomal dysfunction that compromises cell signaling pathways. Deposition of sphingolipids occurs in the autonomic nervous system, dorsal root ganglia, kidney epithelial cells, vascular system cells, and myocardial cells, resulting in organ failure. This manuscript will review the molecular pathogenetic pathways involved in Anderson–Fabry disease and in its organ damage. Some studies reported that inhibition of mitochondrial function and energy metabolism plays a significant role in AFD cardiomyopathy and in kidney disease of AFD patients. Furthermore, mitochondrial dysfunction has been reported as linked to the dysregulation of the autophagy–lysosomal pathway which inhibits the mechanistic target of rapamycin kinase (mTOR) mediated control of mitochondrial metabolism in AFD cells. Cerebrovascular complications due to AFD are caused by cerebral micro vessel stenosis. These are caused by wall thickening resulting from the intramural accumulation of glycolipids, luminal occlusion or thrombosis. Other pathogenetic mechanisms involved in organ damage linked to Gb3 accumulation are endocytosis and lysosomal degradation of endothelial calcium-activated intermediate-conductance potassium ion channel 3.1 (KCa3.1) via a clathrin-dependent process. This process represents a crucial event in endothelial dysfunction. Several studies have identified the deacylated form of Gb3, globotriaosylsphingosine (Lyso-Gb3), as the main catabolite that increases in plasma and urine in patients with AFD. The mean concentrations of Gb3 in all organs and plasma of Galactosidase A knockout mice were significantly higher than those of wild-type mice. The distributions of Gb3 isoforms vary from organ to organ. Various Gb3 isoforms were observed mainly in the kidneys, and kidney-specific Gb3 isoforms were hydroxylated. Furthermore, the action of Gb3 on the KCa3.1 channel suggests a possible contribution of this interaction to the Fabry disease process, as this channel is expressed in various cells, including endothelial cells, fibroblasts, smooth muscle cells in proliferation, microglia, and lymphocytes. These molecular pathways could be considered a potential therapeutic target to correct the enzyme in addition to the traditional enzyme replacement therapies (ERT) or drug chaperone therapy.

## 1. Background

Anderson–Fabry disease (AFD) is a rare X-linked inborn error of glycosphingolipid catabolism that results from mutations in the alpha-galactosidase A gene (GLA) at Xq22 [1].

GLA is a homodimeric glycoprotein that hydrolyses the terminal alpha-galactosyl moieties from glycolipids and glycoproteins. This enzyme predominantly hydrolyzes ceramide trihexoside, and it can catalyze the hydrolysis of melibiose into galactose and glucose. *α*-Galactosidase A (GLA) catabolizes glycosphingolipids with terminal*α*-galactosyl groups. These glycosphingolipids primarily include globotriaosylceramidebut are also present in galabiosylceramide and group B blood antigen.

AFD is caused by an abnormal glycosphingolipid metabolism due to the lack or absence of lysosomal α-galactosidase A activity. From this alteration derives a progressive accumulation of globotriaosylceramide (Gb3) and its deacylated form globotriaosylsphingosine (lyso Gb-3) in the affected cells of various organs. The main cellular structures involved in this accumulation are represented by endothelial cells, epithelial cells, pericytes, myocardial cells, ganglion cells and smooth muscle cells [1].

AFD can be considered a multi-organ systemic disease as various studies have shown that its main pathological action derives from endothelial damage [2]. This pathology was first described in 1898 by Fabry [3] and Anderson [4]. The latter represented a picture of angiokeratoma corporis diffusum universal in males. The pathogenesis of AFD has been fully described [5,6].

Progressive cardiovascular, cerebrovascular and renal dysfunction is related to the accumulation of Gb3 and lyso-Gb-3 [7]. Furthermore, studies show that Lyso-Gb3 induces an inflammatory and fibrogenic response and promotes the development of proteinuria resulting in chronic kidney disease (CKD) and the development of end-stage renal disease (ESRD) [8,9].

Anderson–Fabry disease is a glycosphingolipidosis characterized by the progressive accumulation of glycosphingolipids and the deposition of myelin-like cell inclusions in lysosomes. The alpha-galactosidase A enzyme is found in lysosomes and acts to remove alpha-bonded galactosyl residues from ceramide trihydroxide, causing the catabolism of globotriaosylceramide and related glycosphingolipids [10,11,12].

In Anderson–Fabry disease, the deficiency of the α-galactosidase A leads to an accumulation of Gb3, of its metabolite, globotriaosylsphingosine (Lyso-Gb-3), and of its precursor metabolite called Gal-Gal-Cer in the lysosomes [11].

The abnormal accumulation of Gb3 and Lyso-Gb3 causes cellular dysfunction, resulting in the activation of a pathogenetic pathway which is responsible for progressive damage to multiple organs. Hemizygotic males with AFD have a refined accumulation of Gb3 in the endothelial cells and in the kidneys of Lyso-Gb3 (smooth muscle cells and podocytes) and heart tissue (including valves, cardiomyocytes, nerves, and coronary arteries) [8,12,13]. Nevertheless, the role of Lyso-Gb3 analogues in AFD cellular pathology is still not fully clear.

**Figure 1:**
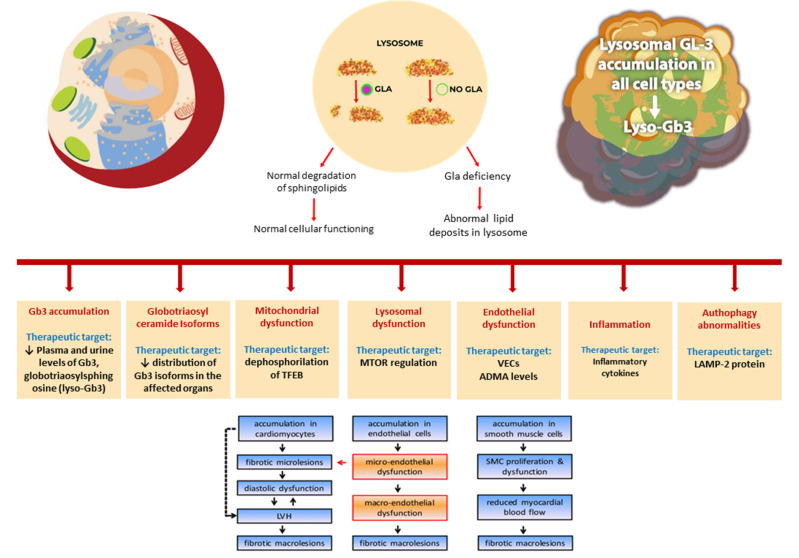
Molecular and cellular alterations involved in pathogenesis of Anderson–Fabry disease.

Molecular pathogenesis of Anderson–Fabry disease encompasses several pathologic mechanisms involving mitochondrial dysfunction, lysosomal dysfunction, GB3 accumulation, globotriaosylceramide isoforms, and globotriaosylsphingosine accumulation and related organ disease, endothelial dysfunction, and autophagy abnormalities (see Figure 1). Several of these pathogenetic molecular abnormalities may represent possible actual and future therapeutic targets.

In this manuscript, we will review the main molecular pathogenetic pathways involved in Anderson–Fabry disease and in its organ damage.

## 2. Mitochondrial Dysfunction in Anderson–Fabry Disease

Deacylated globotriaosylceramide, globotriaosylsphingosine, and a minor additional metabolite are dramatically increased in plasma of classically affected male Fabry patients and the plasma and tissues of Fabry mice. The accumulation of Gb3 causes a lysosomal dysfunction that alters the cell signaling pathway [2,14,15]. Since there is a lysosome–autophagy–mitochondria interaction, the loss of integrity of this interaction is a crucial step in organ damage of AFD [16]. Few studies have evaluated mitochondrial function and energy metabolism in some aspects of FAD pathology [14,15]. The deposition of Gb3 in AFD cardiomyocytes (CM) is responsible for the increased cardiac excitability, with alteration of the calcium activity in the myocardial cells, so as to determine an increased cardiac excitability [17,18].

Myocardial cells, in order to perform their function regularly, need the integrity of the mitochondrial cells. Mitochondrial activity is essential for the integrity of energy metabolism to ensure the typical efficiency of myocardial cells. Machann et al. reported that inhibition of mitochondrial function and energy metabolism plays a significant role in AFD cardiomyopathy [16,19]. Magnetic resonance spectroscopy demonstrated cardiomyocyte involvement and progressive Gb3 storage fibrosis. Some studies have shown a reduction in phosphocreatine, Adenosine-di-phosphate (ADP), Adenosin mono-phosphate (AMP) and adenosine-tri-phosphate (ATP) in left ventricular mass in patients with AFD [16,18]. Furthermore, electron microscopic evaluation of cardiomyocytes demonstrated a reduction in the percentage area of mitochondria in the cytoplasm in proportion to the accumulation of Gb3 [20]. A study showed that the reduction in ischemic tolerance observed in patients with AFD is linked to the increased oxygen requirement due to left ventricular (LV) hypertrophy [20].

Furthermore, progressive lyso-Gb-3 accumulation causes histological damage of kidney cells, resulting from lysosome rupture [9]. Other studies reported that the deposition of Gb3 in podocytes has an essential role in the pathogenesis of glomerular damage [21]. In addition, phagocytosis abnormalities have been documented through the analysis of AFD renal epithelial cells in urine and through evaluation of podocytes in vitro [13]. That may represent a pathogenetic basis for AA renal damage. In AFD, microtubule-associated protein 1A/1B-light chain 3 (LC3-II) upregulation activity, excessive phagocytosis in renal epithelial cells and podocytes, and reduced levels mechanistic target of rapamycin kinase (mTOR) may occur in the kidney [13]. Furthermore, the accumulation of Lyso-Gb3 determines the activation of the inflammatory process in cultured podocytes, as it activates the Notch1 signaling pathway [8,22]. Furthermore, upregulation of Notch1 results in in vivo podocyte lesions and renal fibrosis. As Notch1 signaling pathways act in various mechanisms of energy metabolism, including glycolysis, Krebs cycle, oxidative phosphorylation, and glutamine metabolism [8,23], Notch1 disorders can impair mitochondrial metabolism. In Galt Mtg (CAGE-A4GALT) mice, one study observed that bone marrow cells in the ascending limb had flattened, round mitochondrial ridges [24]. This finding suggests that mitochondrial dysfunction due to Gb3 accumulation can affect mTA1 function and lead to automatic effects, failure of mitochondrial function, and of energy balance. MTOR-dependent signaling pathways [24] represent a critical modulator of both the autophagic–lysosomal fusion process and mitochondrial activity. Overall, these data indicate that impaired sphingolipid metabolism can complicate the mitochondrial function of kidney cells in FD.

MTOR, in its active state, is found on the lysosomal surface. This molecule inhibits autophagy and lysosomal fusion. Amino acid accumulation in the lysosomal lumen can result in the activation of autophagy [25]. Impairment of mTOR activity in fibroblasts and AFD podocytes has been reported [25,26,27]. Other studies have reported that the accumulation of sphingolipid substrates in lysosomes inhibits autophagy–lysosome fusion and disrupts mTOR’s activation/inactivation cycle. ATP models control mTOR activity and mTOR plays an essential role in maintaining the entry of nutrients and growth factors into cells. In response to decreased ATP levels, AMP-activated protein kinase (AMPK) inhibits mTOR activity [28].

Furthermore, mTOR promotes the dephosphorylation of transcription factor EB (TFEB). This molecule also modifies the nuclear structure and the transcription process to trigger autophagy [28,29]. Moreover, mTOR regulates mitochondrial metabolism and promotes the translation of mitochondrial-bound proteins encoded in the nucleus, including mitochondrial transcription factor 1 (Tfam), cytochrome c oxidase (CoxI) subunit 1, and cytochrome c oxidase subunit IV mitochondrial (CoxIV) [29,30]. The activity of cytochrome C oxidase and of the respiratory chain is reduced in fibroblasts of patients with AFD [29,30]. Mitochondrial dysfunction, linked to the impairment of the mTOR pathway, has been evaluated by in vivo studies, which showed that the dysregulation of the autophagy–lysosomal pathway inhibits the mTOR-mediated control of mitochondrial metabolism in AFD cells [29,30].

## 3. Neuropathological Aspects of Gb3 Accumulation in AA

Patients with AFD often have cerebrovascular involvement, with the unusual occurrence of ischemic stroke at a young age [31,32,33] and sometimes chronic cerebrovascular disease with cognitive impairment [34,35,36,37]. Moreover, characteristic findings related to peripheral nerve disease such as typical pain, sensory disturbances, and hypohidrosis have been reported. MRI of the brain shows more infarcts in the brain, cerebellum, and brainstem in many patients with AFD regardless of the presence of neurological signs, and T2 MRI often reveals high-signal areas in the brain’s white matter [34,36,37,38,39,40,41], sometimes very similar to MRI results of demyelinating disease [42].

Cerebrovascular complications are caused by cerebral microvessel stenosis, caused by wall thickening resulting from the intramural accumulation of glycolipids, luminal occlusion, or thrombosis [43,44]. One study reported that globotriaosylceramide metabolism is also defective in central nervous system (CNS) neurons. In fact, this substance does not stain immunohistochemically in the normal brain using a specific antibody [45]. In addition, in AFD, neuronal swelling due to glycolipid accumulation is scarcely present in some restricted nuclei, such as the amygdaloid body, the subiculum, and the dorsal vagus nucleus of the medulla oblongata [44]. The mechanism responsible for the neuronal accumulation of globotriaosylceramide in AFD is not yet fully understood [44].

Regarding the involvement of cerebral vessels in AFD, it was observed that many segments of the subarachnoidal arteries of medium size (Group II: diameter 100–1000 mm) are characterized by having a luminal narrowing caused by intimal fibrosis mixed with smooth muscle cells (SMC), with membrane strongly undulated and stiffened internal elastic due to the total or partial replacement of the medial SMC with fibrosis and adventitial fibrosis [43]. In the case of episodic and progressive heart failure, it is believed that the arterial changes caused by the myocardial accumulation of globotriaosylceramide, typical of the same AFD, can together cause chronic or repeated universal cerebral ischemia resulting in multiple infarcts and axonopathic leukoencephalopathy of the deep cerebral white matter [43,44]. The latter is nourished by medullary arteries characterized by a long course. Thus, moderate to severe multi segmental stenosis and progressive mural stiffening of the medium-sized subarachnoid arteries appear necessary for dementia as a complication of AFD. Furthermore, based on the above, FD dementia is a kind of subcortical vascular dementia and the mechanism of preferential involvement of the subarachnoid arteries of group II is an essential issue to be addressed for the future [43].

## 4. Globotriaosylceramide Isoforms and Globotriaosylsphingosine in Organ Damage Related to Anderson–Fabry Disease

Deacylated globotriaosylceramide, globotriaosylsphingosine, and a minor additional metabolite have been reported as increased in the plasma of classically affected male Fabry patients and plasma and tissues of Fabry mice [45]. Several studies have identified the deacylated form of Gb3, globotriaosylsphingosine (Lyso-Gb3), as the main catabolite that increases in plasma and urine in patients with AFD.

**Figure 2:**
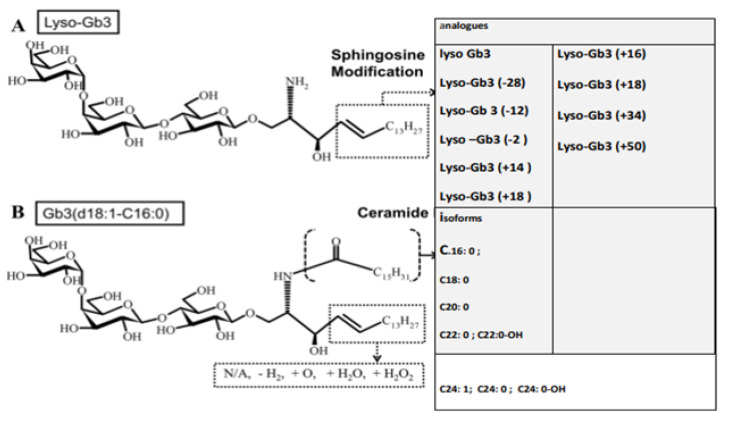
Lyso-Gb3 analogues and Gb3 isoform (A: Lyso GB3; B: Gb3 isoform).

Thus, Lyso-Gb3 analogues having various sphingosine modifications are the biomarkers of the disease (See Figure 2).

Lyso-Gb3 is a valuable biomarker of this disease for diagnosis, the monitoring of disease progression, and the evaluation of therapeutic efficacy [45,46]. This biomarker has analogues with various modifications of sphingosine, and they are also reported as biomarkers of the disease (See Table 1) [9]. Numerous studies have confirmed that such glycosphingolipid accumulation [45,46] is significantly associated with the pathogenesis of Fabry disease. Although Gb3 is a cellular component, its deposit is responsible for endothelial damage and nephropathy through increased cytokine expression. The increase in Lyso-Gb3 also causes injury to glomerular podocytes and sensory neurons and is responsible for the proliferation of smooth muscle cells. This alteration may be related to the vascular defect in Fabry disease. However, to better understand the pathogenesis of Fabry disease, it is necessary to investigate the role of the different species of glycosphingolipids accumulated in the organs and their action on each organ and tissue.

In a study [47], the authors examined the distributions of Gb3 isoforms and Lyso-Gb3 and its analogues in GLA knockout mice using cell cultures, electrophysiology, real-time PCR, immunoblot analysis, Mobility Displacement Electrophoretic Assay (EMSA), and fluorescence microscopy. Gb3 isoforms were examined, having various fatty acids bound to multiple fractions of sphingosine corresponding to Lyso-Gb3 and its four primary analogues (Lyso-Gb3 (−2), Lyso-Gb3 (+16), Lyso-Gb3 (+18) and Lyso-Gb3 (+34)). Ionic fragments derived from the neutral loss of a single galactosyl fragment (162.05 Da) from the precursor ion, not components derived from a ceramide moiety, were analyzed. The authors found that the ceramide portion of the Gb3 isoforms influenced the evolution of several molecular species so that molecules with short, unsaturated, and hydroxylated fatty acids linked to sphingosine developed earlier. The authors found that the Gb3 concentration in the kidneys was higher than that in the heart and liver in both wild-type and GLA knockout mice [47].

The mean concentrations of Gb3 in all organs and plasma of GLA knockout mice were significantly higher than those of wild-type mice [47]. The distributions of Gb3 isoforms vary from organ to organ. Various Gb3 isoforms were observed mainly in the kidneys and kidney-specific Gb3 isoforms were hydroxylated. In knockout mice, the GLA and concentrations of hydrophobic isoforms (e.g., Gb3 (d18: 1-C24: 0)) were higher than those of hydrophilic isoforms Gb3 (e.g., Gb3 (d18: 1-C16): 0) in the organs. However, this was not observed in plasma. Furthermore, in wild-type mice, concentrations of hydrophilic isoforms were high in the plasma [47].

Unlike that of Gb3, the concentration of Lyso-Gb3 was elevated in the liver but not in the kidneys. The mean concentrations of Lyso-Gb3 in all organs and in the plasma of GLA knockout mice were significantly higher than in wild-type mice [47]. The Lyso-Gb3 to Gb3 ratio in plasma was higher than that of organs in both wild-type and GLA-knockout mice. The presence of Lyso-Gb3 was higher than that of its analogues in all organs and in the plasma of wild-type and GLA knockout mice. Among the analogues of Lyso-Gb3, the accumulation of Lyso-Gb3 (−2) and Lyso-Gb3 (+18) were higher than that of the others. During an inflammatory process, endothelial cells (ECs) release autacoids such as NO, prostaglandins, and a factor that causes relaxation through the hyperpolarization of smooth muscle, whereby the vascular diameter is controlled. Studies have shown that the Ca^2+^activated K^+^ channel (KCa3.1) plays an essential role in endothelium–dependent responses during inflammation [47].

**Figure 3:**
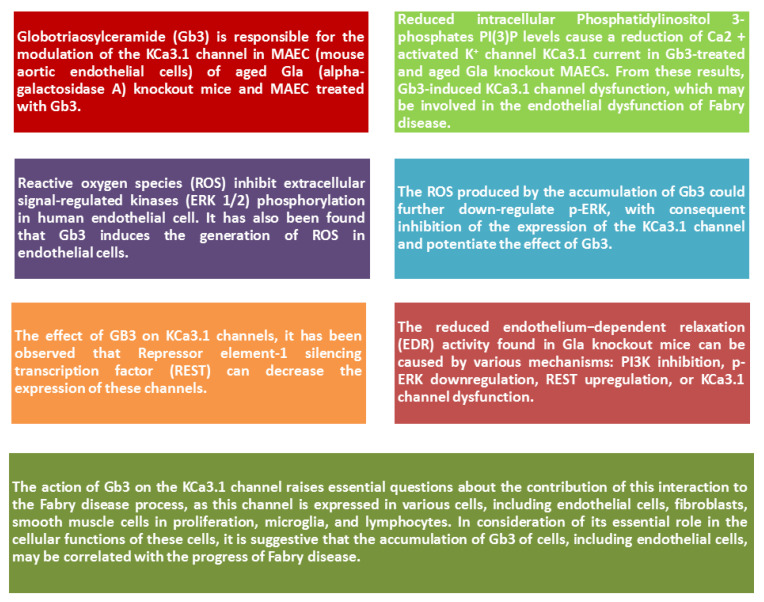
Summary diagram of the role of KCa3.1 channel as a potential target of AFD treatment.

Mutations in KCa3.1 are responsible for impairing endothelial activity and vascular contractility, leading to a predisposition to vascular diseases, including hypertension and atherosclerosis. It emerged that Gb3 is responsible for the modulation of the KCa3.1 channel in MAEC (mouse aortic endothelial cells) of aged Gla knockout mice and MAEC treated with Gb3 [47] (see Figure 3). These results were obtained from the analysis of the expression and activity of the KCa3.1 channel in both MAEC treated with Gla knockout and Gb3. A reduction in protein expression and channel activity was observed in this study. This derives from the fact that Gb3 can inhibit the ERK/AP-1 (Extracellular signal-regulated bkinases) pathway and activate repressor element-1 silencing transcription factor (REST), with a consequent reduction in the expression of KCa3.1 and associated destruction of the channel structures already present. Reduced intracellular PI(3)P (Phosphatidylinositol 3-phosphates) levels cause a reduction ofKCa3.1 current in Gb3-treated and aged Gla knockout MAECs. From these results, Gb3-induced KCa3.1 channel dysfunction, which may be involved in the endothelial dysfunction of Fabry disease, was observed for the first time [47].

In Gla knockout mice, at the cellular level, was observed Gb3 accumulated in the endothelium, analogous to that observed in Fabry disease. This mechanism can be added because reactive oxygen species (ROS) inhibit ERK 1/2 phosphorylation in human endothelial cell. It has also been found that Gb3 induces the generation of ROS in endothelial cells. Therefore, the ROS produced by the accumulation of Gb3 could further down-regulate p-ERK, with consequent inhibition of the expression of the KCa3.1 channel and potentiate the effect of Gb3. Since there is not yet complete clarity in this regard, the role of ROS and Gb3 must be clarified in detail [47,48]. Since inhibition of ERK or PI3K can suppress eNOS expression, the action of Gb3 on down-regulated p-ERK and PI3K could contribute to impaired endothelium–dependent relaxation (EDR) function by reducing NO production, resulting in endothelial damage [47,48].

In addition to the effect of GB3 on KCa3.1 channels, it has been observed that REST can decrease the expression of these channels. In fact, the KCa3.1 channels in endothelial cells regulate the passage of Ca^2+^, thus determining the expression of EDR. Therefore, the compromise of the KCa3.1 channel alters EDR [47,48]. Consequently, the reduced EDR activity found in Gla knockout mice can be caused by various mechanisms: PI3K inhibition, p-ERK downregulation, REST upregulation, or KCa3.1 channel dysfunction. Therefore, in light of the scientific evidence through the use of knockout mice, it was observed that KCa3.1 deficiency has a severe impact on ACh-induced EDR, with a consequent significant increase in arterial blood pressure and an increased risk of atherosclerosis. These results could therefore give an explanation for uncontrolled hypertension in Fabry patients [47,48].

In contrast, the KCa1.1 channel, known as the high conductance Ca^2+^ activated K^+^ channel, is expressed in mouse aortic smooth muscle cells and has been shown to be unaffected by the effect of GB3.

In conclusion, the action of Gb3 on the KCa3.1 channel raises essential questions about the contribution of this interaction to the Fabry disease process, as this channel is expressed in various cells, including endothelial cells, fibroblasts, smooth muscle cells in proliferation, microglia, and lymphocytes. In consideration of its essential role in the cellular functions of these cells, it has been suggested that the accumulation of Gb3 of cells, including endothelial cells, may be correlated with the progress of Fabry disease. Therefore, the KCa3.1 channel could be evaluated as a potential therapeutic target for the disease [46,47,48,49].

## 5. Pathogenesis of Endothelial Dysfunction Linked to Gb3 Accumulation

The link between globotriaosylceramide (Gb3) and globotriaosylsphingosine (lysabilisceramide) and disease severity is not clearly understood. In a study [50], authors examined some Gb3 isoforms (various fatty acids) and Lyso-Gb3 analogs (various sphingosine alterations) in two types of mouse models of Fabry disease: a background a mixed B6/129 or C57BL/6(B6) pure, characterized by prominent cardiac and renal hypertrophy and thermal sensitivity deficit. In the two models, the total levels of Gb3 and Lyso-Gb3 in the heart, kidney, and dorsal root ganglion (DRG) were similar. The C20 fatty acid isoform of Gb3 and some Lyso-Gb3 analogs (+18, +34) showed higher concentrations in Fabry-B6/129 heart tissue than Fabry-B6. There was no difference in the DRG and in the isoforms/analogues of Gb3 and Lyso-Gb3 in the kidneys of the two blocks. The authors also reported that Gb3 significantly deposited in DRG mechanoreceptors, representing a subpopulation of sensory neurons, while still performing functions in Fabry disease. In non-peptidergic nociceptors, they represent a relevant subpopulation for the disease and influence the expression of isolectin-B4 (the marker of non-peptidergic nociceptors) with the role of binding and increasing cell size; no accumulation was detected. Authors also showed that the different degree of deposition of Gb3 or Lyso-Gb3 can play a significant role in the pathogenesis of Fabry disease and that Gb3 and Lyso-Gb3 may not be involved in the pathogenetic damage of AFD in all tissues or structures.

However, endothelial dysfunction represents the main pathological event linking the different clinical symptoms of Anderson–Fabry disease.

Some studies [51,52] conducted on knockout (KO) mice have allowed important insights into the knowledge of the effects of Gb3 accumulation and its relationship with progressive endothelial dysfunction and subsequent vasculopathy.

A study [51] showed the accumulation of Gb3 in the caveolae of aortic endothelial cells. This study evaluated the lipid quantity of primary culture mouse aortic endothelial cell caveolins isolated from null alpha-Gal A to understand the pathogenesis of Fabry disease. From the lipid analysis, it was observed that the excessive accumulation of Gb3 in the endothelial cells of the alpha-Gal in a mouse aorta in culture was deposited at the level of the plasma endothelial membrane. There was also a simultaneous increase in glucosylceramide and lactosylceramide and Gb3 levels in line with age. At the same time, the accumulation of globotriaosylceramide (Gb4) isoform in relation to age was analyzed. At the level of the caveolar membranes, a progressive increase in the concentration of enriched cholesterol and consequent decrease in its levels was also observed, in line with the progressive deposition of Gb3. In this study, inhibition of Gb3 with recombinant human alpha-Gal A protein or d-treo-ethylenedioxyphenyl-P4, a glucosylceramide synthase inhibitor, resulted in increased cholesterol in the caveoles of alpha-deficient mouse aortic endothelial cells. In contrast, recombinant human alpha-gall A failed to normalize cholesterol content. These results demonstrate how the caveolar accumulation of glycosphingolipids in an in vitro model of a lysosomal storage disease is responsible for the changes in the architecture of the lipid microdomains of the plasma membrane and how these lipid alterations may represent one of the possible pathogenetic causes of endothelial damage characteristic of Fabry disease.

Another study [52] used pluripotent stem cells generated from four AFD patients (AFD-iPSC) after induced differentiation into vascular endothelial cells (VECs) to better understand the relationship between Gb3 accumulation and vascular disease. We modified the genome using the Clustered Regularly Interspaced Short Palindromic Repeats (CRISPR)-Cas9 system to eliminate the GLA mutation or eliminate thrombospondin-1 (TSP-1). RNA sequencing was used to analyze global transcriptomes between wild-type (WT) and post-AFD-VEC. The authors reported that excessive modulation of TSP-1 is involved in VEC dysfunction in AFD. The authors observed alterations in angiogenesis in VEC resulting from abnormalities of AFD-iPSC (FD-VEC) even following treatment with recombinant α-galactosidase. Curiously, the FD-VECs produced more p-mothers against decapentaplegic homolog 2 (SMAD2) and TSP-1 than the wild type (WT)-VECs. The authors also observed an increase in TSP-1 levels in the peritubular capillaries of the kidney tissues when performing renal biopsy of patients with AFD. In consideration of the fact that inhibition of SMAD2 signaling or knock out of TSP-1 (TSP-1-/AFD-VEC) protects normal vascular function in AFD-VEC, as well as in gene corrected AFD-VEC, and in light of the fact that excessive oxygen consumption is reduced in the TSP-1-/-AFD-VEC, it was concluded that the overexpression of TSP-1, resulting from the accumulation of Gb3, is more responsible for the observed dysfunction of FD-VEC. These results suggest that reduced VEC angiogenesis in peritubular capillaries may represent one of the possible pathogenetic alterations responsible for the complications.

Therefore, Anderson–Fabry disease is characterized by the early development of vasculopathy and endothelial dysfunction.

A further study [53] set out to better understand the pathological mechanisms of the disease by evaluating serum samples obtained from 17 healthy controls, and 15 AFD patients with and seven without CF. A multiplex ELISA test was performed for 23 different angiogenesis markers in pooled samples. Features showing significant differences between groups were further tested in single models using specific ELISA antibody tests. Symmetric Dimethylarginine (SDMA), l-arginine, asymmetric (ADMA), and l-homoarginine (hArg) were analyzed through liquid chromatography-mass spectrometry. Matrix metalloproteinase 9 (MMP-9) and angiostatin were elevated in patients with AFD compared to controls regardless of the presence of CF. SDMA concentrations were higher in patients with CF. Between L-homoarginine and SDMA (hArg/SDMA) was lower in CF patients than in controls. No significant differences were observed between the l-arginine, hArg and ADMA groups, although there was a trend towards higher ADMA levels and lower hArg levels. A relationship could be identified between renal and cardiac function (*p* = 0.045). The authors concluded that elevated levels of MMP-9 and angiostatin lead to increased extracellular matrix turnover in patients with AFD. Furthermore, the alteration of SDMA and hArg/SDMA was responsible for endothelial dysfunction in these patients.

## 6. Pathogenesis of Lysosomal Damage due to Globotriaosylceramide (Gb3)

The endothelium regulates vascular smooth muscle tone and, therefore, vascular diameter. It is responsible for the pathogenetic basis of perfusion defects and dysfunction of multiple organs, including heart, kidneys, skin, and brain, in individuals with Fabry disease [46].

Endothelial cells release autacoids such as nitric oxide, prostaglandins, and an endothelium-derived hyperpolarizing factor (EDHF) which cause smooth muscle hyperpolarization and relaxation. Intermediate conductance Ca^2+^-activated K^+^ channel (KCa3.1) plays a crucial role in endothelium–dependent responses, including endothelium–dependent hyperpolarization.

The mutation of KCa3.1 causes the alteration of endothelium–dependent control of vascular contractility. This process is responsible for a predisposition to vascular diseases, such as hypertension and atherosclerosis [50,54].

Moreover, some authors observed that in Human embryonic kidney cells (HEK cells), K^+^ channels, expressed by transfection and not endogenously expressed, are degraded. These studies showed that endothelial expression and KCa3.1 activity are reduced by GB3 accumulation and that the defect in the synthesis of this channel protein is the cause of KCa3.1 downregulation [46].

Some authors [49] investigated whether Gb3 induces KCa3.1 endocytosis and degradation. In this study, the authors evaluated the effects of Gb3 on these receptorial structures. The results have shown that Gb3 is responsible for endocytosis and lysosomal degradation of endothelial KCa3.1 via a clathrin-dependent process. This process leads to endothelial damage. In mouse aortic endothelial cells (MAECs) and human umbilical vein endothelial cells treated with Gb3, lysosomal inhibitors seem to inhibit the downregulation of KCa3.1, especially at the level of the plasma membrane. KCa3.1 is not down-regulated by endoplasmic reticulum stress-inducing agents. Gb3 increased the levels of early endosome antigen 1 and lysosomal associated membrane protein 2 in MAECs. In MAECs from aged α-galactosidase A (GLA)-knockout mice, KCa3.1 expression was downregulated. In these cells, early endosome antigen 1 and lysosomal-associated membrane protein 2 expression were upregulated. In MAECs from aged GLA-knockout mice, clathrin was in the cell border and clathrin knockdown determined the recovery of KCa3.1 expression. Rab5, an effector of early endosome antigen 1, was upregulated, and Rab5 knockdown caused regular KCa3.1 expression.

## 7. Molecular Pathogenesis of Renal Involvement in Anderson–Fabry Disease

The cellular mechanisms of kidney dysfunction in Anderson–Fabry Disease are not clearly described. It seems to play a crucial role in kidney injury, one of the programmed necrotic cell death pathways as necroptosis [55]. The diagnosis of AFD is often delayed because of the heterogeneous phenotype, disease severity variability, and symptoms onset. Several studies have shown that patients with rapid progression of kidney disease have higher urinary protein to urinary creatinine ratios [56]. Furthermore, pre-existing diseases or conditions, such as smoking, hyperlipidemia, or hypertension, may contribute to a progressive reduction of GFR [57,58]. However, GLA variants and the environmental factors cannot clearly explain the phenotypic variability of AFD. Often, even between the same family members with the same pathogenic GLA variant, there is enormous clinical variability [58].

Podocytes are terminally differentiated cells, so they do not divide; therefore, their replacement potential in adults is limited. Deposition of glycosphingolipids in podocytes, endothelial cells, and other cell types leads to formation of myelin-like inclusions, which are the hallmark of the disease [59,60]. This accumulation results in the fusion of podocyte foot processes and clinically in advancing proteinuria. Damaged podocytes detach from the glomerular basement membrane lost in the urine. Podocyturia is a representation of the clinical severity of Fabry nephropathy [61]. Podocytes detachment and urinary excretion are correlated with reduced adhesion of podocytes to the extracellular matrix due to low levels of podocalyxin [62]. Podocyturia disrupts glomerular selectivity, causes albuminuria/proteinuria, and leads to glomerulosclerosis and fibrosis [7]. Podocyturia is an excellent marker to predict renal involvement; even before that, the albumin/creatinine ratio (ACR) changes. Indeed, a positive correlation was found between podocyturia and ACR [63]. Despite podocyturia being an early clinical sign of kidney injury and possibly serving as a diagnostic test to assess kidney involvement, it is still not regularly used in clinical practice. This is because methods for assessing podocyturia are not yet standardized and not available in most clinical settings.

Receptor interacting serine/threonine kinase 3 (RIPK3) plays an essential role in necroptosis. Some authors [57] studied the role of protein phosphokinase RIPK3 in the pathogenesis of Fabry nephropathy both in vivo and in vitro. They showed that cell viability of podocytes decreased after Lyso-Gb3 treatment in a dose-dependent manner, with increasing RIPK3 expression. Furthermore, increased reactive oxygen species (ROS) levels caused by Lyso-Gb3 treatment and their alleviation by GSK’872 (a RIPK3 inhibitor) suggested the presence of oxidative stress via an RIPK3-dependent pathway. RIPK3 inhibitor normalized cytoskeleton rearrangement caused by Lyso-Gb3. Injection of Lyso-Gb3 in mice induced increase albuminuria and reduced podocytes counts in the glomeruli. In conclusion, this study indicated a new pathway in Fabry nephropathy. It showed that Lyso-Gb3 caused podocyte loss and subsequent foot process effacement, leading to albuminuria, a clinical manifestation of Fabry nephropathy.

Transforming growth factor-β1 (TGF-β1) and the macrophage inhibitory factor receptor cluster differentiation 74 (CD74) are implicated in the tissue damage of diabetic nephropathy [64]. Globotriaosylsphingosine (Lyso-Gb3) accumulates in the AFD. This bioactive molecule seems to affect the secretion of mediators responsible for glomerular damage and, therefore, could play a role in Fabry disease nephropathy. Some authors [64] hypothesized that Lyso-Gb3 could be accountable for releasing secondary mediators of damage in glomerular podocytes. It has been shown that vitamin D receptor activation has nephroprotective actions in diabetic nephropathy. They also want to study whether these nephroprotective actions might be applied to Lyso-Gb3. In this study, the authors analyzed potential modulation by vitamin D receptor activation of biological activity of Lyso-Gb3 in cultured human podocytes. Paricalcitol or calcitriol activates the vitamin D receptor and, consequently, maintains normal TGF-β1, CD74, and extracellular matrix induced by Lyso-Gb3. The results demonstrated that paricalcitol, which reduces proteinuria in diabetic nephropathy, has the same action in Fabry nephropathy. In fact, paricalcitol causes the interruption of the harmful pathway activated by Lyso-Gb3 in podocytes. The authors concluded thatLyso-Gb3 could be responsible for glomerular injury in Fabry disease, modulating the release of secondary mediators of glomerular injury common to diabetic nephropathy. Paricalcitol has a preventive action on this injury pathway activating VDR. This study suggests that paricalcitol could have a potential adjunctive role in Fabry nephropathy therapy.

In renal impairment in Fabry disease, podocyturia causes glomerulosclerosis and is responsible for kidney disease progression. It is known that podocyte attachment to the glomerular basement membrane involves integrins [63,64]. Some authors studied Lyso-Gb3 actions on integrins in Fabry nephropathy. The αvβ3 integrin, in addition to UPAR, causes podocyte detachment and podocyturia. The authors wanted to study if Lyso-Gb3 modulates αvβ3 expression in podocytes. For this aim, they [65] evaluated in cultured human podocytes how Lyso-Gb3 affects the mRNA expression of some genes, in particular the Integrin Subunit Alpha V (*ITGAV)* and Integrin transforming growth factor beta 1 (*ITGB3)*, encoding integrins αv and β3. The results showed that *ITGAV* and *ITGB3* mRNA levels were increased in the presence of Lyso-Gb3. The same pattern of gene expression was already detected for PLAUR (UPAR). However, these findings are in contrast with the subsequently found increase in levels of proinflammatory cytokines and other markers of podocyte stress (CD80, TGFβ1, CD74, Notch1, and HES). Glycolipid overload induces human podocytes stress; the expression of components of the αvβ3/UPAR system is increased in Fabry nephropathy, but this is in contrast with a tardive increased expression of other mediators of podocyte injury. This suggests a possible therapeutic strategy in which the αvβ3/UPAR system may be a target in Fabry nephropathy.

Lyso-Gb3 is a glycolipid that accumulates in serum in Fabry disease and determines increased extracellular matrix synthesis in podocytes. Some authors [8] studied, in cultured human podocytes, how Notch1 responds to Lyso-Gb3 stimulation. High concentrations of Lyso-Gb3, stimulating in podocytes the activation of Notch1 signaling, determine increased levels of Notch1 and HES1. HES1 is upregulated in response to Lyso-Gb3. This upregulation is inhibited by a specific Notch1 small interfering RNA (siRNA) or γ-secretase inhibitor. The same action was detected on upregulation of Notch1, Notch ligand Jagged1, and chemokine (monocyte chemoattractant protein-1 (MCP1), Regulated upon Activation, Normal T Cell Expressed and Presumably Secreted (RANTES) expression. NFκB contributes to the activation of Nocht1-mediatedinflammatory response. Notch1 siRNA prevents the Lyso-Gb3-induced activation of nuclear factor kappa B (NFκB), and the NFκB inhibitor prevents Lyso-Gb3 action on chemokine upregulation. Furthermore, it was observed [66] that in podocytes, Notch1 induces a fibrogenic response and Notch siRNA inhibits Lyso-Gb3 activity on fibronectin mRNA. These data are supported by the presence of different molecules, as such active Jagged1, HES1 and Notch1, in Fabry kidney biopsies. The authors concluded that Lyso-Gb3 is a promoter of a Notch1-mediated inflammatory process, and it stimulates fibrogenic responses in podocytes; these Lyso-Gb3 mediated responses may have a role in the pathogenesis of Fabry nephropathy.

Genetic modifiers associated with the specific clinical presentation of Anderson–Fabry Disease have not yet been evaluated with genome-wide association studies (GWAS). Some studies reported the association of variants of the gene involved in inflammatory and thrombosis/hemostasis mechanisms with a higher risk of cerebral lesions and stroke in Fabry patients [8,67,68]. Alcohol Dehydrogenase 4 (ADH4) (rs1126670, rs1126671, rs2032349) and Alcohol Dehydrogenase 5 (ADH5) (rs2602836), gene variants in human alcohol dehydrogenase family genes, have been related to AFD progression [66]. Some authors hypothesized that some gene modifiers are likely to contribute to organ involvement and disease progression as the clinical presentation itself varies widely between family members with identical pathogenic GLA variant. Another field of investigation is the analysis of transcriptomics focusing on some selected urinary miRNA characterized by a higher or lower degree of expression in AFD. A decrease in certain miRNA species, such asmiR-29 and miR-200, has been associated with renal fibrosis prior to the onset of pathological albuminuria [69]. Some serum miRNAs such asmiR-1307-5p, miR-21-5p, miR-152-5p, and miR-26a-5p have been reported as significantly down-regulated in Anderson–Fabry patients. Furthermore, miR-19a-3p and miR-486-5p have been reported to be down-regulated in subjects with AFD [70].

Concerning epigenomics, the real burden of DNA and/or histone modifications on the AFD progression and particularly on nephropathy remains unclear. Few studies analyzed epigenetic changes in AFD [71,72]. In Fabry patients, high concentrations of the methylated/non-methylated Gb3 isoform were found in urine compared to controls characterized by the absence of trace amounts of the methylated Gb3 isoform in control urine samples [73].

A study [73] evaluated non-methylated Gb3 isoforms normalized to creatinine. Authors studied these biomarkers in Fabry patients compared to healthy controls and evaluated correlations between biomarker urinary excretion and age, gender, treatment, and genotype of patients. They analyzed, with a tandem mass spectrometer, urine samples from 150 Fabry patients and of 95 healthy controls. The authors reported some significant correlations between Gb3 isoform concentrations, gender, and treatment. In five patients with the late-onset cardiac mutation, p.N215S was found abnormal concentrations of methylated Gb3 isoforms compared to their non-methylated homologues.

Glycosphingolipid biomarkers, such as globotriaosylceramide (Gb_3_) isoforms, globotriaosylsphingosine (Lyso-Gb3) and related analogues, and galabiosylceramide (Ga_2_) isoforms and analogues were found to be abnormally increased in urine and plasma of Fabry patients; these molecules could be used as specific biomarkers of the disease. Another study [74] reported that the methylated Gb_3_isoforms are particularly useful for screening Fabry patients who present late-onset cardiac variant mutations and renal variants.

## 8. Molecular Pathogenesis of Cardiac Involvement in Anderson–Fabry Disease

Cardiac involvement is a common finding in Anderson–Fabry disease, both in hemizygous men and heterozygous women, and it also represents a significant cause of morbidity and mortality. Accumulation of globotriaosylceramide regards various heart cells, including conduction system cells, valvular fibroblasts, cardiomyocytes, endothelial cells of heart vessels, and vascular smooth muscle cells. The typical finding of cardiac involvement of AFD is cardiac hypertrophy that may worsen, developing an impairment of contractility and diastolic filling impairment. Other possible cardiac involvement signs are atrioventricular conduction disturbances, coronary insufficiency, arrhythmias, and valvular involvement.

Gb_3_ storage by itself is not enough to explain the degree of cardiac hypertrophy, conduction abnormalities and other cardiac manifestations. In a patient with Fabry disease with a remarkably increased cardiac mass, the autopsy showed a low contribution (1–2%) of the accumulated material to severe heart hypertrophy [75]. Some studies reported that that storage induces progressive lysosomal and cellular malfunctioning that, in turn, activates common signaling pathways leading to hypertrophy, apoptosis, necrosis, and fibrosis. Energy depletion has recently been proposed as a possible pathogenetic background in numerous metabolic and even sarcomeric hypertrophic cardiomyopathies, including Fabry disease [76]; in fact, impairment of energy management was observed in skin fibroblasts [77]. It seems that, in addition to mechanical storage, biochemical factors might also play a role. In patients with neuronal ceroid lipofuscinosis, another lysosomal storage disease has been described as mitochondrial disfunction, so some authors examined mitochondrial function in fibroblasts from patients with AFD [78]. This study showed a significant reduction of respiratory chain enzymes I, IV, and V activities in AFD-cells. The mitochondrial marker enzyme citrate synthase activity demonstrated that mitochondrial recovery was normal, and cellular protein content was not significantly different. ADP and AMP concentrations were substantially lower in AFD-cells. ATP was slightly but not significantly reduced. Thus, it seems reasonable that organ damage in AFD may not only be explained by mechanical storage of glycosphingolipids, but lysosomal storage material may be responsible for mitochondrial dysfunction with a reduction of respiratory chain enzyme activities and subsequent lower levels of energy-rich phosphates.

Inflammation might play a critical role in the development of cardiac changes in Fabry disease. In end-stage cardiomyopathy in patients with Fabry disease, fibrosis in the left ventricle (but not in the right ventricle) is a common finding [77,78]. A study evaluated cardiomyocyte dysfunction in patients with Fabry disease and cardiomyopathy. The authors showed that intracellular accumulation of GB3 is associated with oxidative damage of proteins and DNA, which leads to cardiomyocyte dysfunction and death; this study also revealed the presence of hypertrophic and disorganized cardiomyocytes, cells death through apoptosis, high expression of inducible nitric oxide synthase and nitrotyrosine, and evidenced glycosphingolipid accumulation in endomyocardial biopsies [79]. Moreover, these results showed that several cytokines levels were increased in the serum of Fabry patients. Among these wereIL-1β, TNF-α, IL-6, monocyte chemoattractant protein-1 (MCP-1), intercellular adhesion molecule-1, and soluble vascular adhesion molecule [80]. These findings indicate that proinflammatory cytokines might play a role in the progression of Fabry disease-related cardiomyopathy. The functions of proinflammatory cytokines in cardiomyopathy may differ between patients with and without Fabry disease.

The autopsy from patients with Fabry disease showed various apoptotic myocytes on caspase-3-positive cytoplasmic staining, and in the myocardium, there was also a mild T-lymphocyte interstitial infiltrate [81]. Inflammatory macrophages seem to have an essential role in myocardial injury; they were observed in some endomyocardial biopsies from patients with Fabry disease. Moreover, the same study reported Gb3 accumulation in myocytes of both atria and ventricles and in endothelial cells, coronary arteries, aorta, valve tissue, and smooth muscle cells. The presence of extensive areas of myocyte disarray associated with fibrosis has also been reported [80,81].

## 9. Molecular Mechanisms and Possible Therapeutic Targets

α-Gal gene is a 12kb comprising seven exons, located on the X chromosome (Xq22.1), and its mutation causes Fabry disease. Over 100 different mutations have been described up to now. Among these, most are missense mutations but insertions, deletions, and RNA processing defects caused by aberrant splicing have also been reported [82]. These mutations are responsible for reduced or absent enzyme activity.

Glycosphingolipids such as globotriaosylceramide (Gb3) are components of the plasma membrane that are degraded in the lysosome. Several hydrolyzing enzymes, including α-Ga, are necessary for the correct catabolism of glycosphingolipids. The progressive accumulation of the incompletely degraded substrate Gb3 in the cells is due to a deficiency of this hydrolase [82]. The deposits lead to cellular dysfunction or degeneration; GB3 is accumulated within multi vesicular bodies or intracytoplasmatic masses. It seems that intracellular Gb3 storage triggers a series of pathological mechanisms correlated to the progressive impairment of the respective tissue and organ function with severe consequences. Recent advances in molecular biology and genetic engineering have enabled the development of causal therapies for storage diseases [82]. A possible therapeutic strategy is supplying the deficient human enzyme, which is replaced by infusion of recombinant enzyme preparation. Two randomized trials of enzyme replacement therapy in Fabry disease have been conducted [83]. Both enzyme therapies were well tolerated and seemed to be effective in catabolizing the lipid deposits. Using agalsidase β, the authors showed reductions of capillary endothelial cell Gb3 storage in the heart. There were no functional effects reported; thus, the clinical relevance of these results is unknown. Schiffmann et al. [83], using agalsidase α, showed a significant decrease in QRS duration, suggesting a reduction in left ventricular hypertrophy. Preliminary data of further studies in Mainz and London using agalsidase α confirmed these findings. They showed a significant decrease in left ventricular mass and an increase in diastolic function and cardiac output [84].

Chemical chaperones are molecules that bind and stabilize misfolded proteins in the endoplasmic reticulum (ER), thereby stimulating regular trafficking and contributing to increased residual activity [85]. This subclass of therapy is being developed for a range of disease, including Anderson–Fabry Disease, Gaucher disease, Pompe disease, Batten disease, mucopolysacchirodosis type IIIC, and GM1 andGM2 gangliosidosis [86]. Migalastat [87] has been evaluated in both ERT-naïve and ERT-treated A patients with treatment resulting in increased and sustained endogenous α-GAL activity, with the maintenance of kidney function and a reduction in the left ventricular mass index [88,89]. Reduction in Lyso-Gb3 levels of up to 45% has been demonstrated in ERT-naïve adult patients. However, not all patients experienced an increase in activity of the enzyme and a reduction of Lyso-Gb3 levels [90]. This raises the caveat that this form of therapy exerts efficacy only in specific mutations of AFD. It is estimated that only 35–50% of AFD mutations are amenable to migalastat use with367 amenable and 711 non-amenable mutations [91].

Substrate reduction therapy (SRT) acts on the downstream effects of enzyme deficiency by reducing substrate production resulting from the defective enzyme before accumulation, thereby reducing synthesis to a level compatible with residual clearance [92]. Given the mechanism of action, these therapies have particular promise in lysosomal storage disorders (LSD). GCS inhibitors limit conversion of ceramide to glycosphingolipid, eventually leading to a reduction of Gb3 production. Lucerastat (N-butyl deoxy galactonojirimycin), an imino-sugar inhibitor of GCS, lowered Gb3 in kidneys of Fabry mice null for α-GAL [93]. No severe adverse effects were observed in two separate randomized, double-blind, placebo-controlled, single and multiple ascending dose studies on lucerastat [94]. A subsequent open-label, randomized study of 10 patients with AFD who received lucerastat at 1 g twice daily demonstrated a mean reduction of 55% in Gb3 levels, along with being well-tolerated over 12 weeks [93].

## 10. Autophagy Abnormalities in Molecular Pathogenesis of Anderson–Fabry Disease

Degradation of metabolic wastes and damaged cell elements such as mitochondria inside the cell is a critical process to maintain cellular homeostasis and respond to environmental stress. Macro autophagy or autophagy is a cell process that sequesters cytoplasmic cargos into double-membrane compartments (autophagosomes) and then directs them towards lysosomes [95]. Defects in autophagy process may cause the accumulation abnormal materials in tissues, and this abnormality seem to be the pathogenestic basis of some human cardiovascular diseases [25,95]. Lysosome-associated membrane protein 2 (LAMP-2) is the crucial mediator of the process of interplay between autophagy process and lysosomes.

Furthermore, one study reported how a well-functioning lysosome/autophagic pathway is fully involved in the vascular architecture and function [25]. In Anderson–Fabry disease, ischemic stroke represents an expression of organ damage of the disease and pathological studies in AFD patients reported that VSMC proliferation is induced in cerebral arteries and that there is a relationship between autophagy defects and small vessel disease [25,95]. In animal models of VSMC autophagy-related 7 knockout mouse Atg7^−/−^, some authors reported an increase in medial thickening of the aorta due to VSMC hypertrophy 15.

A study [96] analyzed the biological features of LAMP-2-deficient mice and cultured cells. LAMP-2-deficient mice at 9–24 months of age showed medial thickening with luminal stenosis due to proliferation of vascular smooth muscle cells (VSMC) in muscular arteries. Ultrastructural analysis of VSMC revealed various autophagic vacuoles scattered throughout the cytoplasm, suggesting impaired autophagy of long-lived metabolites and degraded organelles (i.e., mitochondria). These findings indicate that LAMP-2 deficiency leads to arterial medial hypertrophy with the phenotypic conversion of VSMC, resulting from age-dependent accumulation of cellular waste generated by aberrant autophagy. These pathogenetic mechanisms could also be involved in vascular complications of Anderson–Fabry Disease.

A study [97] by means of an experimental mouse model of Fabry disease, alpha-galactosidase A deficiency, studied brain pathology in mice models of AFD aiming to explain the real role of the autophagy–lysosome pathway. Authors reported that alpha-galactosidase A-deficient mouse brains showed a high density of punctate perinuclear immunoreactivity for the autophagy marker microtubule-associated protein light-chain 3 (LC3) in the parenchyma of several brain regions and increased vascular immunoreactivity for lysosome-associated membrane protein-1 (LAMP-1). Ultrastructural analysis also showed endothelial cell inclusions with electron densities and a pronounced accumulation of electron-dense lipopigment [97]. These findings indicate widespread neuropathology and focused axonal neurodegeneration in alpha-galactosidase A-deficient mouse brain in association with disruption of the autophagy–lysosome pathway and may offer the basis for future mechanistic assessment of the contribution of the autophagy–lysosome pathway to neuropathologic phenotype of AFD [96].

The role of autophagy disorders in AFD does not only involve neurological complications of the disease but it also regards the development of progressive renal damage. Histological studies suggest that the accumulation of Gb3 in podocytes plays an important role in the pathogenesis of glomerular damage. Some authors [22] studied a human podocyte model of Fabry’s disease by combining RNA interference technology with lentiviral transduction of human podocytes. Knockdown of a-galactosidase A expression resulted in diminished enzymatic activity and slowly progressive accumulation of intracellular Gb3. Interestingly, these changes were accompanied by an increase in autophagosomes as indicated by an increased abundance of LC3-II and a loss of mTOR kinase activity, a negative regulator of the autophagic machinery. These data suggest that dysregulated autophagy in a-galactosidase A-deficient podocytes may be the result of deficient mTOR kinase activity. This finding links the lysosomal enzymatic defect in Fabry’s disease to deregulated autophagy pathways and provides a promising new direction for further studies on the pathomechanism of glomerular injury in Fabry patients.

## 11. Conclusions

Anderson–Fabry disease is an X-linked lysosomal storage disease caused by absent or reduced α-galactosidase A activity and the consequent accumulation of GB3 in endothelial cells [98]. GB3 accumulation is associated with several clinical signs and symptoms, such as cardiac problems, renal failure, and central nervous system pathology [98]. The location of lyso-GSL in plasma is unclear. Lyso-Gb3 was detected associated with albumin but not in lipoproteins [13]. Furthermore, α-galactosidase-A activity is inhibited by Lyso-Gb3, increasing GB3 storage. Lyso-Gb3 also affects smooth muscle cell proliferation; other studies suggested that globotriaosylsphingosine stimulates cardiomyocytes and vascular smooth muscle cell proliferation [99]. A study conducted on human podocytes hypothesized that Lyso-Gb3 could have a role in the pathogenesis of Fabry nephropathy [64]. Lyso-Gb3 affects the production of TGFβ, invariant chain (CD74), and extracellular matrix (fibronectin and type IV collagen). These components are involved in the fibrotic response, which leads to kidney failure [64]. In the plasma of Fabry patients, Lyso-Gb3 was shown to inhibit NO synthase. This could contribute to the vasculopathy and cardiac symptoms in Fabry patients [100,101]. These effects are contrasted by paricalcitol or calcitriol, which activates the vitamin D receptor. Another study demonstrated, using human podocytes, that Lyso-Gb3 also induced inflammatory response. The γ-secretase inhibitor IX (GSI IX) could inhibit activation of NF-κB, derived by the Notch1 signaling pathway. This pathway induced monocyte chemoattractant protein-1 (MCP-1/CCL2) in human podocytes [8]. Furthermore, in DC, monocytes and Peripheral blood mononuclear cells (PBMCs) from Fabry patients showed high levels of pro inflammatory cytokines. Some authors studied GB3 in monocyte-derived dendritic cells and macrophages, adding GB3 and simultaneously inhibiting α-Gal. From these results, GB3 seems to be a toll like receptor 4 (TLR4) recognized ligand [102]. Preventing GB3 clearance led to an increased production of IL-1β and TNFα. Furthermore, blocking TLR4 with antibodies reduced the cytokine induction mediated by GB3 [102]. However, it is not yet clear if (lyso-)GB3 also has a modulating action on immunity via type II NKT cells [101,103,104].

Considering that lyso-GSL could be toxic, it would be interesting to attempt to reduce the levels of this molecule. A way to achieve this result would be to reduce their accumulation with substrate reduction therapy (SRT). In conclusion, in addition to future gene therapies to correct the enzyme defect, SRT is a potential therapeutic strategy, as well as the traditional enzyme replacement therapies (ERT) or drug chaperone therapy (PCT) [105].

## Figures and Tables

**Table 1:** Gb3 isoforms and Lyso-Gb3.

Kidney-Specific Gb3 (Globotriaosylceramide) Isoforms	Cardiac Specifics Gb3 Isoforms	Lyso-Gb3 Analougues
Gb3 (d18: 1–C24: 0)	Gb3 (d18: 1–C16: 0)	Lyso-Gb3 (−2)
Gb3 (d18: 1–C16): 0)		Lyso-Gb3 (+16)
		Lyso-Gb3 (+18)

## Abbreviations

AFD	Anderson Fabry disease
Gb3	Globotriaosylceramide
GLA	Alpha-galactosidase A
ESRD	End-stage renal disease
ADP	Adenosine-di-*phosphate*
AMP	Adenosin mono-*phosphate*
ATP	adenosine-tri-phosphate
LC3-II	Microtubule-associated protein 1A/1B-light chain 3
MTOR	Mechanistic target of rapamycin kinase-
AMPK	AMP-activated protein kinase
TFEB	Transcription factor EB
Tfam	Mitochondrial transcription factor 1
CoxI	Cytochrome c oxidase subunit 1
Cox IV	Cytochrome c oxidase subunit IV
ECs	Endothelial cells
KCa3.1	Ca^2+^ activated K^+^ channel
MAEC	Mouse aortic endothelial cells
PI(3)P	Phosphatidylinositol 3-phosphates
ROS	Reactive oxygen species
ERK 1/2	Extracellular signal-regulated kinases
VECs	Endothelial cells
EDR	Endothelium–dependent relaxation
CRISPR)-Cas9	Clustered Regularly Interspaced Short Palindromic Repeats Cas 9
TSP-1	Thrombospondin-1
SMAD2	p-mothers against decapentaplegic homolog 2
SDMA	Symmetric Dimethylarginine
ADMA	l-arginine, asymmetric
hArg	l-homoarginine
MMP-9	Metalloproteinase 9 Pathogenesis of lysosomal damage due to globotriaosylceramide (Gb3)
EDHF	Endothelium-derived hyperpolarizing factor
KCa3.1	Ca^2+^-activated K^+^ channel
HEK cells	Human embryonic kidney cells
ACR	Albumin/creatinine ratio
RIPK3	Receptor Interacting Serine/Threonine Kinase 3
TGF-β1	Transforming growth factor, beta 1
CD74	cluster differentiation 74
ITGAV	Integrin Subunit Alpha V
ITGB3	transforming growth factor beta 3,
PLAUR (UPAR)	Plasminogen Activator, Urokinase Receptor (rokinase plasminogen activator surface receptor)
MCP1	Monocyte chemoattractant protein-1
RANTES	Regulated upon Activation, Normal T Cell Expressed and Presumably Secreted
REST	Repressor element-1 silencing transcription factor
GWAS	genome-wide association studies
ADH4	Alcohol Dehydrogenase 4
ADH5	Alcohol Dehydrogenase 5
LAMP-2	Lysosome-associated membrane protein 2
VSMC	Vascular smooth muscle cells
GSI IX	γ-secretase inhibitor
PBMCs	Peripheral blood mononuclear cell
TLR4	Toll-like Receptor 4
SRT	substrate reduction therapy
